# Supplementary material for: Parenting interventions to promote early child development in the first three years of life: A global systematic review and meta-analysis
Source: PLoS Med. 2021 May 10;18(5):e1003602. doi: 10.1371/journal.pmed.1003602 (PMC8109838; doi:10.1371/journal.pmed.1003602)
Supplement: S1 Protocol — (DOCX) [file pmed.1003602.s002.docx]

**S1 Protocol. Final review protocol.**

The current review was initially conceptualized as two separate reviews: the first review aimed to examine the impact of responsive caregiving (RC) interventions on child development outcomes, and the second sought to assess the impact of interventions to support early learning (EL) on child development outcomes. As a result, two separate protocols were originally submitted for the two specific types of parenting interventions:

- *RC Protocol*: Responsive caregiving interventions: a systematic review and meta-analysis. PROSPERO 2018 CRD42018092458 Available from: <https://www.crd.york.ac.uk/prospero/display_record.php?ID=CRD42018092458>
- *EL Protocol:* Interventions to support early learning: a systematic review and meta-analysis. PROSPERO 2018 CRD42018092461 Available from: <https://www.crd.york.ac.uk/prospero/display_record.php?ID=CRD42018092461>

The current review applies a broader definition of parenting interventions and examining the effects of any type of parenting intervention on child development and caregiving outcomes as part of this single review. The table below indicates any changes that were made from the two original prospectively registered protocols and applied in the final protocol/current review.

| Preregistered PROSPERO Protocols | Final Protocol Used in the Current Review |
| --- | --- |
| **Primary Research Questions** | |
| *RC Protocol:* The objective of this review is to synthesize the evidence surrounding the role of responsive caregiving interventions for improving early child development (ECD) outcomes.  *EL Protocol:* The objective of this review is to synthesize the evidence surrounding the role of interventions for caregiving to support early learning on improving early childhood development (ECD) outcomes. | The current review combines the two research objectives (which had originally been separated) and takes a more expansive approach to review in one study the effectiveness of any type of parenting intervention delivered during the first three years of life on ECD. |
| **Search Strategy** | |
| *Both RC & EL Protocols:* The literature search will be performed across five electronic bibliographic databases: Embase, PubMed, PsycINFO, Cochrane Central Register of Controlled Trials (CENTRAL), and ERIC. Search strategies were developed in accordance with each database. A string of keywords was determined to capture four broad categories: (1) caregiving interventions, (2) targeting children aged 0-3 years, (3) randomized controlled study design, and (4) assessed an ECD outcome. Keywords were informed by search terms and keywords used in prior systematic reviews related to caregiving interventions, as well as through consultations with a research librarian to select relevant MeSH terms and search filters. Reference lists of relevant studies and reviews will also be reviewed for any additional studies that are not retrieved from the main search. No geographic, temporal, or language constraints will be imposed. | Six electronic bibliographic databases were searched, including Embase, MEDLINE, PsycINFO, CINAHL, Web of Science and Global Health Library. The search was last updated to include publications up to November 15, 2020. |
| **Types of studies to be included** | |
| *Both RC & EL Protocols:* We will only include randomized controlled trials with a group sample size greater than 85. The sample size restriction of N>85 was selected as this is the sample size required to find a medium effect size on the Bayley scales of d=0.50, or half a standard deviation difference between the group (Aboud & Yousafzai, 2015). | The sample size restriction was removed. |
| **Condition or domain being studied** | |
| *RC Protocol:* Impact of responsive caregiving interventions on early child development outcomes.  *EL Protocol:* Impact of interventions to support early learning on early child development outcomes. | We no longer focus exclusively on these two specific types of parenting interventions, but instead we more comprehensively include any type of parenting intervention. |
| **Participants/population** | |
| *Both RC & EL Protocols:* Inclusion Criteria: Studies of either RC or EL interventions that have enrolled caregivers (caregiver, defined as the legal guardian, biological parent, or adult responsible for the well-being of the child) and their young children. Interventions will be included if: a) the sample mean age of children at enrollment into the intervention was <36 months, or b) the intervention targeted women who were pregnant at enrollment. Exclusion Criteria: Interventions will be excluded if they: a) targeted children who were preterm or had chronic illness, low birth weight, or clinical diagnosis of disability or behavioral disorder, b) enrolled children older than 36 months of age (on average), or c) were restricted to caregivers with an illness or disability. However, preventive interventions targeting children and caregivers at risk for mental health conditions, developmental delays, or behavioral problems will be included. | No changes. |
| **Intervention(s), exposure(s)** | |
| *RC Protocol:*  Inclusion criteria: Intervention that focused on promoting responsive caregiving (see original protocol for definition). Exclusion criteria: (i) Interventions that did not focus on promoting positive caregiver-child interactions.  *EL Protocol:*  Inclusion criteria: Interventions that focused on supporting early learning for young children (see original protocol for definition).  Exclusion criteria: (i) Interventions that did not focus on supporting early learning. | The current review focused on any type of parenting intervention (which includes interventions that focus on responsive caregiving, early learning, and other parenting components). We applied a more expansive definition of parenting interventions as those that aim to improve caregiver interactions, behaviors, knowledge, beliefs, attitudes, or practices with their children in order to improve ECD. See S1 Table for final definitions of parenting interventions, including responsive caregiving interventions. |
| **Comparator(s)/control** | |
| *Both RC & EL Protocols:* A control group that received either, 1) a variant of the same caregiving intervention (e.g., one that did not include Responsive Caregiving components (*RC protocol*) or components to support early learning (*EL protocol*), 2) a different caregiving intervention, or 3) no intervention/care as usual/waitlist control. | No changes. |
| **Main outcome(s) & Measures of effect** | |
| *Both RC & EL Protocols:*  *Main outcome:* Early child development outcomes relating to: Cognition; Communication and Language; Socio-Emotional Skills; and/or Fine and Gross Motor Skills. *Measures of effect:* Evaluation time point at the completion (or the closest assessment following the completion) of the full intervention (which includes any booster sessions). | Two additional domains of ECD were examined in the current review: behavior problems (e.g., internalizing and externalizing problems) and attachment. |
| **Additional outcome(s)** | |
| *RC Protocol:*  I. Any other measured child outcomes, such as child growth and nutrition indicators, secure attachment, and other related outcomes; II. Change in caregiver-level outcomes, such as child-caregiver interactions, quality of the home environment, responsive caregiving behaviors and activities (talk, play, health-seeking behaviors), knowledge of ECD, emotional wellbeing, and any other measured caregiver-level outcomes.  *EL Protocol:*  I. Any other child outcomes, such as competencies targeted by early learning activities (compliance, skill mastery, sustained attention, play skills), child growth and nutrition, and other related outcomes; II. Change in caregiver-level outcomes, such as child-caregiver interactions, quality of the home environment, caregiving behaviors and activities (talk, play, health-seeking behaviors), knowledge of ECD, emotional wellbeing, and any other measured caregiver-level outcomes. | The current review focuses on four measures of parenting as the secondary outcomes: parenting practices, knowledge, parent-child interactions, and parental depressive symptoms. Attachment was examined as a primary outcome as a domain of ECD. Child nutrition, growth and health outcomes were not examined. |
| **Data extraction (selection and coding)** | |
| *Both RC & EL Protocols:* All titles of studies from the search results will be screened independently by two members of the research team. All relevant abstracts will be reviewed and filtered based on the inclusion/exclusion criteria outlined above. Full texts of relevant articles will then be reviewed, again applying the inclusion/exclusion criteria outlined above. Data from studies that meet the inclusion/exclusion criteria will be extracted by one of seven graduate research assistants using a standardized, pre-piloted form. Types of quantitative and qualitative data to be extracted include:  1. Details related to sample characteristics.  2. Details related to intervention characteristics.  3. Details related to risk of bias.  4. Data necessary for effect size calculation for all outcomes of interest (means, standard deviations, sample sizes of pre- and post-scores in intervention and control groups).  In order to ensure standardization of the data extraction process the discrepancies that arise will be resolved through regular group discussions with the team leads. For quality assurance/quality control, 50% of the first half of retrieved studies will undergo a second data extraction by an independent coder for reliability. 20% of second half of retrieved studies will undergo a second data extraction by an independent coder. | No changes. |
| **Risk of bias (quality) assessment** | |
| *Both RC & EL Protocols:* Each individual study selected for the review will be assessed for risk of bias using the criteria outlined by the Cochrane Risk of Bias Assessment Tool for randomized controlled trials recommended by the Cochrane Handbook version 5.1.0. The risk of bias in the included studies will be assessed alongside the data extraction process by considering the following characteristics: (a) Randomization sequence generation; (b) Concealment of allocation to treatment group; (c) Blinding of participants and investigators; (d) Reporting of data on all study participants (taking into account attrition and exclusions); (e) Complete reporting of all study outcomes that were specified a priori; (f) Other sources of bias (including considerations around measurement reliability and validity). Disagreements between the team members over the risk of bias in particular studies will be resolved by group discussion and consensus. | No changes. |
| **Strategy for data synthesis** | |
| *Both RC & EL Protocols:* A detailed description of how all data for each outcome were handled will be provided, including a justification for the approach taken for each outcome. The final review will include a narrative synthesis of the findings from the included studies, structured around the content and delivery of intervention, target population characteristics, and types of child and caregiver outcomes. Results from the systematic review will be presented in tables. A meta-analysis will be conducted if the number of available studies is sufficient. This will depend on the level of homogeneity in intervention contents, theoretical objectives, comparison conditions, and outcome measures across studies. Quantitative data synthesis will be conducted with either fixed effect or random effects meta-analysis based on a test of heterogeneity. Heterogeneity will be evaluated using Cochran's Q test of heterogeneity and the I² statistic. Subgroup analyses will be conducted to identify potential sources of heterogeneity using random-effect meta-analysis methods to estimate the average effect across groups of studies. Results from the meta-analyses will be displayed in tables and with a forest plot. The Egger regression asymmetry test for publication bias will be calculated for the outcomes to assess the possibility of publication bias. | No changes. |
| **Analysis of subgroups or subsets** | |
| *Both RC & EL Protocols:* If the necessary data are available, patterns of effect will be reviewed across predetermined subgroups, which may potentially include:  - Targeted vs. universal programs.  - Child characteristics: Child age at enrollment.  - Select program characteristics: Intended dosage; Interventions delivered in groups vs. individually vs. combination; Number and type of behavior change techniques used in an intervention; Type of provider (e.g., community health worker, clinical health provider, community lay person/volunteer, teacher, researcher, NGO staff). | Subgroup analyses were conducted by:  - Country-income level  - Average child age  - Interventions that included a component to enhance responsive caregiving  - Intervention duration  - Delivery modality  - Program setting  - Study quality score |
