# Supplementary material for: Parenting interventions to promote early child development in the first three years of life: A global systematic review and meta-analysis
Source: PLoS Med. 2021 May 10;18(5):e1003602. doi: 10.1371/journal.pmed.1003602 (PMC8109838; doi:10.1371/journal.pmed.1003602)
Supplement: S1 Text — (DOCX) [file pmed.1003602.s003.docx]

**S1 Text. Search strategy.**

(Infant[Mesh] OR "Child, Preschool"[Mesh] OR infant[tiab] OR infants[tiab] OR infant’s[tiab] OR neonate[tiab] OR neonates[tiab] OR neonatal[tiab] OR newborn*[tiab] OR new born*[tiab] OR baby[tiab] OR babies[tiab] OR toddler[tiab] OR toddlers[tiab] OR toddlerhood[tiab] OR preschool*[tiab] OR pre school*[tiab] OR early childhood[tiab] OR young children*[tiab] OR "Perinatal Care"[Mesh] OR perinatal[tiab] OR antenatal[tiab] OR ante natal[tiab] OR postnatal[tiab] OR post natal[tiab] OR age 0[tiab] OR aged 0[tiab] OR age zero[tiab] OR aged zero[tiab] OR age 1[tiab] OR aged 1[tiab] OR age one[tiab] OR aged one[tiab] OR age 2[tiab] OR aged 2[tiab] OR age two[tiab] OR aged two[tiab] OR 1 year old*[tiab] OR one year old*[tiab] OR 2 year old*[tiab] OR two year old*[tiab] OR 3 year old*[tiab] OR three year old*[tiab] OR 3 years of age[tiab] OR 2 years of age[tiab] OR 1 year of age[tiab] OR under 2 years[tiab] OR under 1 year[tiab]) AND ("Parenting"[Mesh] OR "Child Rearing"[Mesh] OR "Maternal Behavior"[Mesh] OR "Parent-Child Relations"[Mesh] OR "Parents"[Mesh] OR "Caregivers"[Mesh] OR parents[tiab] OR parenting[tiab] OR mother[tiab] OR mothers[tiab] OR maternal behav*[tiab] OR parental behav*[tiab] OR paternal behavior[tiab] OR parent infant[tiab] OR infant parent[tiab] OR father[tiab] OR fathers[tiab] OR caregiv*[tiab] OR care giv*[tiab] OR child rearing[tiab]) OR parent child[tiab] OR child parent[tiab] OR parent training[tiab] OR parent education[tiab] OR parental training[tiab] OR parental education[tiab] AND ("Child Behavior"[Mesh] OR "Child Development"[Mesh] OR "Cognition"[Mesh] OR "Executive Function"[Mesh] OR "Emotional Intelligence"[Mesh] OR "Emotions"[Mesh] OR "Motor Skills"[Mesh] OR attachment[tiab] OR attention[tiab] OR behavior[tiab] OR behavioral[tiab] OR behaviors[tiab] OR behaviour[tiab] OR behavioural[tiab] OR behaviours[tiab] OR child development[tiab] OR cognition[tiab] OR cognitive[tiab] OR communication[tiab] OR communicative[tiab] OR compliance[tiab] OR conduct problem*[tiab] OR executive function*[tiab] OR emotional[tiab] OR emotions[tiab] OR empathy[tiab] OR fine motor[tiab] OR language[tiab] OR mastery[tiab] OR motivation[tiab] OR motor skill*[tiab] OR peer relation*[tiab] OR play skills[tiab] OR prosocial[tiab] OR reading[tiab] OR social[tiab] OR socialization[tiab] OR socio emotion*[tiab] OR socioemotion*[tiab]) AND random*[tw] AND English[lang]
