# Supplementary material for: Parenting interventions to promote early child development in the first three years of life: A global systematic review and meta-analysis
Source: PLoS Med. 2021 May 10;18(5):e1003602. doi: 10.1371/journal.pmed.1003602 (PMC8109838; doi:10.1371/journal.pmed.1003602)
Supplement: S1 Table — (DOCX) [file pmed.1003602.s004.docx]

**S1 Table. Definition of parenting interventions and those that focus on responsive caregiving versus those that do not.**

| **Parenting interventions** | |
| --- | --- |
| Parenting interventions are programs intended to improve a core set of interconnected behaviors, attitudes, knowledge and skills of parents to optimally promote early child development [1]. Parents are defined as the legal guardian, biological parent, or adult responsible for the well-being of the child [2]. Other interventions that focus on caregiving to improve child health or nutrition, without a specific objective to also promote early child development, are not classified as parenting interventions for the purposes of this review. | |
| **Interventions that focus on responsive caregiving** | **Interventions that do not focus on responsive caregiving** |
| Responsive caregiving interventions aim to enhance caregiver-child interactions by promoting caregivers’ responsiveness, or care that is prompt, consistent, contingent, and developmentally appropriate to the child’s cues, signals, and needs [3]. For example, responsive caregiving interventions may seek to support parents with regards to the following behaviors: facilitating parental attunement and identification of the child’s needs, following the child’s lead, helping the child to focus, supporting the child’s exploration, and scaffolding development through contexts which may include play, communication, or feeding [4]. Interventions that relate to parenting more generally without a focus on promoting responsive caregiver-child interactions – or any study that does not adequately describe the intervention goals or activities to discern whether it focused on responsive caregiving – are not classified in this category. | These interventions may instead more generally focus on caregiving competencies to promote early child development or increased opportunities for early learning and development. However, these interventions do not include direct components to enhance responsive caregiver-child interactions and relationships. Examples of interventions in this category may include caregiver peer group meetings; home-visiting programs to improve caregiver awareness of developmental milestones and developmentally appropriate activities for young children; book or toy sharing interventions; or sessions providing advice on other aspects of parenting such as positive discipline, routines, feeding, and child health and development. Studies that do not clearly specify intervention goals or activities to determine whether they include components of responsive caregiving are classified in this category. |

**References**

1. Black MM, Walker SP, Fernald LCH, Andersen CT, DiGirolamo AM, Lu C, et al. Early childhood development coming of age: science through the life course. Lancet (London, England). 2017;389(10064):77-90. doi: 10.1016/S0140-6736(16)31389-7.

2. Britto PR, Ponguta LA, Reyes C, Karnati R. A systematic review of parenting programmes for young children in low-and middle-income countries. New York, NY: United Nations Children’s Fund, 2015.

3. Eshel N, Daelmans B, de Mello MC, Martines J. Responsive parenting: interventions and outcomes. Bull World Health Organ. 2006;84(12):991-8. Epub 2007/01/24. doi: 10.2471/blt.06.030163.

4. Black MM, Aboud FE. Responsive Feeding Is Embedded in a Theoretical Framework of Responsive Parenting. J Nutr. 2011;141(3):490-4. doi: 10.3945/jn.110.129973.
