# Supplementary material for: Parenting interventions to promote early child development in the first three years of life: A global systematic review and meta-analysis
Source: PLoS Med. 2021 May 10;18(5):e1003602. doi: 10.1371/journal.pmed.1003602 (PMC8109838; doi:10.1371/journal.pmed.1003602)
Supplement: S2 Table — (DOCX) [file pmed.1003602.s005.docx]

**S2 Table. Measures used for each ECD and parenting outcome domain.**

| **Outcome domain** | **Measure** | **Number of studies using the measure** |
| --- | --- | --- |
| Cognitive development | Bayley Scales of Infant and Toddler Development | 33 |
|  | Griffiths Scales of Child Development | 7 |
|  | Stanford-Binet Intelligence Scales | 4 |
|  | Ages and Stages Questionnaire | 3 |
|  | Preschool Developmental Assessment Scale | 2 |
|  | Kaufman Assessment Battery for Children | 2 |
|  | McCarthy Scales of Children's Abilities | 2 |
|  | INTERGROWTH-21st Neurodevelopmental Assessment | 1 |
|  | Peru Wawa Wasi National Programme - ECD instrument | 1 |
|  | Wechsler Preschool and Primary Scale of Intelligence | 1 |
|  | Schedule of Growing Skills | 1 |
|  | Developmental Profile-II | 1 |
|  | Cattell Scales | 1 |
|  | Bracken School Readiness Assessment | 1 |
|  | Mullen Scales of Early Learning | 1 |
|  | Ages and Stages Questionnaire: Inventory | 1 |
|  | Checklist created by authors | 1 |
| Language development | Bayley Scales of Infant and Toddler Development | 13 |
|  | MacArthur-Bates Communicative Development Inventory | 7 |
|  | Peabody Picture Vocabulary Test | 4 |
|  | Griffiths Scales of Child Development | 4 |
|  | Preschool Language Scale | 4 |
|  | Ages and Stages Questionnaire | 3 |
|  | Preschool Developmental Assessment Scale | 2 |
|  | Peru Wawa Wasi National Programme - ECD instrument | 1 |
|  | Early Language Milestone | 1 |
|  | Stanford-Binet Intelligence Scales | 1 |
|  | Developmental Profile-II | 1 |
|  | Gesell Development Schedules | 1 |
|  | Mullen Scales of Early Learning | 1 |
|  | Infant Babbling using LENA Recording | 1 |
|  | Language Behaviors | 1 |
|  | Ages and Stages Questionnaire: Inventory | 1 |
|  | Wechsler Preschool and Primary Scale of Intelligence | 1 |
|  | Communication and Symbolic Behavior Scale | 1 |
|  | Denver-II | 1 |
|  | Caregiver reported measure designed by authors | 1 |
| Motor development | Bayley Scales of Infant and Toddler Development | 22 |
|  | Griffiths Scales of Child Development | 5 |
|  | Ages and Stages Questionnaire | 3 |
|  | Saving Brains Early Child Development Scale | 1 |
|  | Peru Wawa Wasi National Programme - ECD instrument | 1 |
|  | Developmental Test of Visual-Motor Integration | 1 |
|  | Developmental Profile-II | 1 |
|  | Gesell Development Schedules | 1 |
|  | Mullen Scale of Early Learning | 1 |
|  | Ages and Stages Questionnaire: Inventory | 1 |
|  | Denver-II | 1 |
|  | Caregiver reported measure designed by authors | 1 |
| Socioemotional development | Ages and Stages Questionnaire | 5 |
|  | Ages and Stages Questionnaire: Social-Emotional | 4 |
|  | Brief Infant–Toddler Social and Emotional Assessment | 4 |
|  | Bayley Scales of Infant and Toddler Development | 3 |
|  | Strength and Difficulties Questionnaire | 3 |
|  | Behavior Assessment System for Children | 2 |
|  | Infant–Toddler Social and Emotional Assessment | 2 |
|  | Bayley Behavior Rating Scale | 1 |
|  | Wolke's Scale | 1 |
|  | Social Skills Rating System | 1 |
|  | Peru Wawa Wasi National Programme - ECD instrument | 1 |
|  | 3 box coding scale | 1 |
|  | Adaptive Social Behavioral Inventory | 1 |
|  | Gesell Development Schedules | 1 |
|  | Ages and Stages Questionnaire: Inventory | 1 |
|  | Denver-II | 1 |
| Behavioral development | Child Behavior Checklist | 16 |
|  | Brief Infant–Toddler Social and Emotional Assessment | 3 |
|  | Infant–Toddler Social and Emotional Assessment | 2 |
|  | Behavior Assessment System for Children | 2 |
|  | Strength and Difficulties Questionnaire | 2 |
|  | Eyberg Child Behavior Inventory | 1 |
|  | Devereux Early Childhood Assessment | 1 |
|  | Parenting Stress Index-Difficult Child | 1 |
|  | Child Behavior Development Scale | 1 |
|  | Infant Behavior Questionnaire | 1 |
| Attachment | Ainsworth Strange Situation Procedure | 5 |
|  | Waters' Attachment Q Set | 3 |
|  | Attachment Q-Sort | 1 |
|  | Toddler Attachment Sort-45 | 1 |
|  | Alarm Distress Baby Scale | 1 |
|  | Parent Attachment Diary | 1 |
|  | Child–Adult Relationship Experimental Index Toddlers | 1 |
| Parenting knowledge | Questionnaire created by authors | 14 |
|  | Raising a Baby | 1 |
|  | Knowledge of Infant Development Inventory | 1 |
| Parenting practices | Home Observation Measurement of the Environment | 22 |
|  | Family Care Indicators | 8 |
|  | Parenting practices questionnaire developed by authors | 3 |
|  | Stim-Q | 2 |
|  | Caldwell Home Scale | 1 |
|  | Alabama Parenting Questionnaire | 1 |
|  | Coded video observations | 1 |
| Parent-child interactions | Observations of interactions (no specific name for tool) | 16 |
|  | Nursing Child Assessment Satellite Training | 5 |
|  | Landry Parent–Child Interaction Scales | 2 |
|  | Mother & Baby Interaction Scale | 2 |
|  | Nursing Child Assessment Teaching Scale | 1 |
|  | Observation of Mother-Child Interactions | 1 |
|  | Responsive Talk | 1 |
|  | Dyadic Parent–Child Interaction Coding System | 1 |
|  | Reciprocity during book sharing interactions | 1 |
|  | Global Ratings Mother-Infant Relationship | 1 |
|  | Maternal Interaction Structured Situation | 1 |
|  | LENA audio files of interactions | 1 |
|  | Audio recordings of dialogic reading behaviors | 1 |
| Parental depressive symptoms | Center for Epidemiologic Studies Depression Scale | 14 |
|  | Beck Depression Inventory | 5 |
|  | Self-Reporting Questionnaire | 2 |
|  | Major Depression Inventory | 2 |
|  | Edinburgh Postnatal Depression Scale | 1 |
|  | General Health Questionnaire | 1 |
|  | Patient Health Questionnaire | 1 |
|  | Questionnaire created by authors | 1 |
