# Supplementary material for: Parenting interventions to promote early child development in the first three years of life: A global systematic review and meta-analysis
Source: PLoS Med. 2021 May 10;18(5):e1003602. doi: 10.1371/journal.pmed.1003602 (PMC8109838; doi:10.1371/journal.pmed.1003602)
Supplement: S1 Fig — (DOCX) [file pmed.1003602.s006.docx]

**S1 Fig.** **Risk of bias across all randomized controlled trials.**
