# Supplementary material for: Parenting interventions to promote early child development in the first three years of life: A global systematic review and meta-analysis
Source: PLoS Med. 2021 May 10;18(5):e1003602. doi: 10.1371/journal.pmed.1003602 (PMC8109838; doi:10.1371/journal.pmed.1003602)
Supplement: Attachment — Submitted filename: Response to editors comments.docx [file pmed.1003602.s008.docx]

**Requests from Editors:**

1. Abstract: Methods and Findings: Please include p-values in addition to 95% CIs where applicable for all main results presented in the Abstract.

- This has been done.

2. In-text citations: Throughout the text, please do not include spaces within brackets for references, for example please use [2,3] rather than [2, 3].

- This has been done.

3. Methods: Thank you for indicating that your review was preregistered with PROSPERO and providing the information. You note two registrations associated with the review, it would be helpful if you could include as a supporting information file the final review protocol used, noting any changes from the prospectively registered protocol.

- We have included a new supporting information file (entitled “S1 Protocol”) that presents the final review protocol used and indicates where any changes during our process of combining the two originally separate protocols into a single more expansive review (the present study).

4. Results: Page 14: Please clarify if the pooled result for infant-caregiver attachment was significant “The pooled result showed a moderate positive impact on improving attachment (SMD=0.29, 95% CI: 0.18, 0.40; I2=0%, P=0.92; Fig 7).”

- We have added the P-value (P<0.001) in addition to the 95% CI associated with the pooled effect. The second P-value corresponds to the I^2^ statistic for heterogeneity.

5. Results: Page 16: Please clarify if the pooled result for parental depressive symptoms was statistically significant: “The pooled result did not indicate a significant reduction in caregiver depressive symptoms (SMD=-0.07, 95% CI: -0.16, 0.02; I2=76%, P<0.001; Fig 11).”

- We have added the P-value (P=0.08) in addition to the 95% CI associated with the pooled effect. The second P-value corresponds to the I^2^ statistic for heterogeneity.

6. Results: Page 17: Please clarify as moderator analyses were not done for behavioral development or attachment: “Although moderator results for the other outcomes were not significantly different by country income level, the magnitudes of the effects were all consistently greater in LMICs versus HICs.”

- On pg. 19, we have edited this sentence by specifying the outcomes we are referring to: “Although effects on child socioemotional development, parenting knowledge, parent-child interactions, and parental depressive symptoms did not significantly differ by country-income level, the magnitudes of the estimates were consistently greater for all outcomes in LMICs versus HICs (Table 3).”

7. Results: Page 17: Please clarify these results are also presented in Tables 2 and 3: “However, there were no other consistent differences in outcomes by child age, intervention duration, delivery, setting, or study risk of bias score.”

- On pg. 19, we have indicated that these results can be found in Tables 2 and 3: “…there were no other subgroup differences in any outcomes by child age, intervention duration, delivery, setting, or study risk of bias score (Tables 2 and 3).”

8. Discussion, Page 19: Please add a space between the word and reference bracket in this sentence: “While bundling multiple components together (e.g., mental health and parenting) to create intervention packages is recommended in the literature [5]...”

- This has been done.

9. Discussion, Page 21: Please revise to: “Our statistically non-significant moderation results by child age are consistent…” and “Furthermore, our statistically non-significant moderation results by program duration, delivery modality, and setting are also consistent with…”

or similar to clarify “non-significant” as “not statistically significant” in these sentences.

- We have revised all instances where we had written “non-significant” to “not statistically significant” as suggested.

10. Table 2 and Table 3: Please define the abbreviation (SMD) in the legend.

- This has been done.

11. Page 42: Please remove the Conflicts of Interest and Funding sections, and ensure all information is accurately entered in the “Competing Interests” and “Financial Disclosure” section of the manuscript submission form as this information would be published alongside the article.

- This has been done.

12: Page 43: Please remove the “Human Participant Protection” statement and ensure that all relevant information pertaining to the ethical approval of your study is included in the Methods.

- This has been done.

13. References: Please double check the consistency of all references in the list, including abbreviations and capitalizations for journal names. For example, “The Lancet Global Health” and “The Journal of nutrition” should be “Lancet Glob Health” and “J Nutr” and please check all throughout. Please similarly format the references listed in S1 Table.

- This has been done.

14. Figures 2-11: Please give a descriptive legend to describe each figure, including all symbols, bars, and values, and including an X axis label. Please provide the p value associated with the SMD and 95% CIs at least for the overall effects reported.

- We have included X axis labels in each of the forest plots and have provided descriptive legends to each figure caption. In the manuscript text, we have provided the p-values associated with all pooled effect sizes.

15: S1 Table: Please use brackets rather than superscript for reference numbers.

- This has been done.
