# Supplementary material for: Parenting interventions to promote early child development in the first three years of life: A global systematic review and meta-analysis
Source: PLoS Med. 2021 May 10;18(5):e1003602. doi: 10.1371/journal.pmed.1003602 (PMC8109838; doi:10.1371/journal.pmed.1003602)
Supplement: Attachment — Submitted filename: Response to reviewer comments.docx [file pmed.1003602.s007.docx]

**Editors comments:**

1. **Regarding the paper format/style - please structure your abstract using the PLOS Medicine headings (Background, Methods and Findings, Conclusions) -writeup in each section should use full sentences rather than bullet-point type sentence fragments. In the last sentence of the Abstract Methods and Findings section, please include a brief note about any key limitation(s) of the study's methodology. Finally please also head up the first section of the paper as "Introduction".**

- This has been done.

2. **At this stage, we ask that you include a short, non-technical Author Summary of your research to make findings accessible to a wide audience that includes both scientists and non-scientists. The Author Summary should immediately follow the Abstract in your revised manuscript. This text is subject to editorial change and should be distinct from the scientific abstract. Please see our author guidelines for more information:** [**https://journals.plos.org/plosmedicine/s/revising-your-manuscript#loc-author-summary**](https://journals.plos.org/plosmedicine/s/revising-your-manuscript#loc-author-summary)

- This has been done.

3. **If possible please reformat the citation style into PLOS Medicine's format (should be straight forward if using referencing software) - this should use callouts formatted as sequential numerals in square brackets (not superscript).**

- This has been done.

4. **The reviewers have noted that the date for search cutoff was May 2019, which now makes the systematic review a little dated. The editors felt that updating the search to the present would be ideal in order to address this concern.**

- We have updated the search to include publications up to the search cutoff of November 15, 2020. From this updated search, we identified and added to our review 11 new eligible articles representing 11 unique RCTs. (We also included 1 new study based on one eligible article shared by Reviewer #2.) So, in total, this revised manuscript includes 12 new RCTs. Generally speaking, our results remained robust and did not substantially change with the addition of the new studies. We have updated the Methods and Results accordingly in text, as well as all the results tables and figures.

5. **We noted that although the authors state the paper was reported with the use of the PRISMA reporting tool, we did not see a completed PRISMA checklist in the supplementary files - please download the word or pdf version of the checklist at** [**http://www.prisma-statement.org/PRISMAStatement/Checklist.aspx**](http://www.prisma-statement.org/PRISMAStatement/Checklist.aspx)**. When completing the checklist (which should be included as part of the supplementary materials), please use section and paragraph numbers, rather than page numbers.**

- We have uploaded the completed PRISMA checklist as part of the supplementary materials (file: S1 PRISMA Checklist).

6. **In addition reviewers noted that an omission in the systematic review methods was the lack of consideration of publication bias (or other possible forms of bias affecting the array of effects across studies), please note that a component of PRISMA is the assessment of risk of bias across studies (items 15 and 22). See the PRISMA paper (elaboration and explanation) at** [**https://journals.plos.org/plosmedicine/article?id=10.1371/journal.pmed.1000100#s6**](https://journals.plos.org/plosmedicine/article?id=10.1371/journal.pmed.1000100#s6) **which sets out what can be reported and analysed under those points.**

- On pg. 9 in the Methods section, we have added details about conducting Egger’s regression tests to assess risk of bias in the estimated pooled effects across studies for each outcome analyses. On pg. 12 in the Results section, we have reported the results of the Egger’s statistical tests.

**Reviewer #1:**

**This paper describes the carrying out of a systematic review and meta-analysis of parenting interventions to promote early child development in the first three years of life. This is an important review in bringing together in one place a large body of evidence. It will therefore be a helpful reference in the future to locate research in this field. I have a few comments that may help to improve the usefulness of this paper.**

1. **Abstract
   The outcomes included have a list that includes "…behaviour development..". Should this read "..behaviour, development.." (i.e. is there a missing comma?) or does this refer to behaviour and the development of behaviour. Please clarify. Also check that this list is consistent with later repeated version of the list which subtly differ.  Please check and amend as necessary.**

- In the listing of outcomes, we had initially stated “behavior development” so that the word “development” could refer to each of the domains (cognitive, language, motor, socioemotional, and behavior) without having to repeat the word “development” with each domain. However, we agree that this is confusing in addition to the fact that as the reviewer notes we used the term interchangeably with “behavior problems” elsewhere in the manuscript. We believe “behavior problems” is clearer and have revised accordingly throughout the manuscript.

2. **Introduction
   The introduction is important for readers to build up a sense of the literature on the subject.
   The sentence in para 2 of the introduction beginning "Parenting interventions can encompass a range of interventions including…."  will need improved referencing. I would suggest using systematic reviews or theoretical papers to lay out the types of parenting interventions available.**

- In the second paragraph of the Introduction, we have added examples of other types of parenting interventions with references to prior systematic reviews.

3. **Also there are some big categories missing such as parental mental health, interventions to develop parental sensitivity etc. Please check the literature and make sure you include the main categories so the reader can orientate to the review.**

- In the second paragraph of the Introduction, we have added the importance of parental mental health interventions and parental sensitivity interventions (with references to prior systematic reviews) as part of our overview of main types of parenting interventions.

4. **In para 3 the authors say that a review published in 2018 is "relatively outdated". I hope that we can still draw some information from a review published in the last ten years, so I would perhaps remove this or use different terminology. Perhaps keep to the focus on what you have done differently, which is clear.**

- This clause has been removed.

5. **Methods
   I would usually expect to see criteria laid out in the usual PICOS criteria way. Is there a reason you have not done this?**

- Our descriptions of the study selection/eligibility criteria as well as the details outlined in the Outcomes and Data analysis sections (pgs. 6-7) cover all the components of PICOS. Our reporting style is consistent with many other recent systematic reviews and meta-analyses published in *PLOS Medicine* and in the literature on reviews of parenting interventions and other social and behavioral interventions.

6. **I think it would have been stronger if you had not excluded children with disability or parents with disability. Please discuss this limitation in the relevant section.**

- With respect to parenting programs, our focus was parenting programs that offered universal support for all caregivers and young children rather than targeted or indicated support for caregiver and children with additional risks and needs who require specialized services. In particular, parenting programs for caregivers of children with disability may be integrated with specific rehabilitation services for children with specific conditions and employ specialized measures of developmental progress making outcomes (e.g., McConachie et al., 2001; McConkey & Samadi, 2013) which very much warrants its own systematic review as discussed in Yousafzai et al., *Arch Dis Child* (2014). Among universal parenting programs, most studies have either excluded enrollment for children with disabilities to the intervention program (or excluded for the research evaluation component) making it difficult to ascertain information about the inclusiveness of programs and benefits to children with disabilities. On pg. 23, we have added a statement to the limitations section of the manuscript.

7. **Based on your exclusion criteria it would seem that if an intervention targeted 0-7 year old children it would be excluded as the average age would be 3.5 years. Is this correct? If so please discuss this as a limitation. This may rule out some long term interventions for example.**

- The reviewer is correct. On pg. 23 in the Limitations section, we have added this point about the child age inclusion/exclusion criterion and its implications to our study findings.

8. **Were the graduate research assistants trained (please state)? Please discuss this. Is this a limitation? For example, using multiple graduates could lead to some bias.**

- At the bottom of pg. 6/top of pg. 7 of the Methods, we have explained how all graduate research assistants were trained and that the data extraction process included independent reviewers to minimize bias. More specifically, the research assistants’ data extractions were all independently completed by a second reviewer, and furthermore by a third reviewer in a random subsample of 35% of articles.

9. **Results
   Could you say something about what proportion of studies used validated outcome measures? (As opposed to observation/frequency counts/bespoke measures etc.). This may be important information for future researchers.**

- In the Results section, as part of each paragraph for the different outcomes, we have added the proportion of studies that mentioned any details regarding the reliability or validity of the measures as used in the included articles/study populations. Overall, this information was not consistently reported across studies. The specific measures used across studies for each outcome domain are listed in S2 Table. We have also elaborated on this point on pg. 23 in the Limitations section and have highlighted the need for more of this information in future research.

10. **Discussion
    You have highlighted shortcomings of studies in terms of quality and bias. It would be helpful to have a very brief paragraph outlining the particular aspects future studies need to do/improve upon to generate better evidence. You have done this to an extent. I think this is very important aspect of this paper.**

- On pg. 24 to the end of the Discussion, we have added a new paragraph extending upon how future trials could generate better quality evidence with less risk of bias.

11. **In the discussion you say that effects in LMICs are larger than in HICs. Can you clarify if any studies have directly compared the same intervention programme in those two settings or is it the case that the different studies use different interventions but show larger effect sizes. If the latter can you draw this conclusion? The different intervention used could be a factor for example.**

- We agree for this important point. On pg. 20 of the Discussion, we have added this clarification and caveat.

12. **The limitations section is well written although please include the things I have mentioned above for thoroughness.**

- We have address all the reviewer’s points in the revised Limitations section (pgs. 22-24).

13. **In several of the areas mentioned there are existing systematic reviews that I would have expected to be referenced as the main systematic reviews in place prior to this one.
    These for example include:
    Behaviour
    Moon, D. J., Damman, J. L., & Romero, A. (2020). The effects of primary care-based parenting interventions on parenting and child behavioral outcomes: A systematic review. Trauma, Violence, & Abuse, 21(4), 706-724.

    Attachment
    Wright, B., Hackney, L., Hughes, E., Barry, M., Glaser, D., Prior, V., ... & Garside, M. (2017). Decreasing rates of disorganised attachment in infants and young children, who are at risk of developing, or who already have disorganised attachment. A systematic review and meta-analysis of early parenting interventions. Plos one, 12(7), e0180858.

    etc. I think these should be in an introductory paragraph to set the context of this review so that the reader can look at the existing evidence. Here you can include these existing systematic reviews and reference them. This sets the scene for what is known and what this review adds, and helps people from different fields navigate the literature. This would greatly enhance the paper.**

- As part of the second paragraph of the Introduction, we have elaborated upon the prior systematic reviews of different types of parenting interventions (including behavior and attachment interventions) and have referenced these prior systematic reviews shared by the reviewer.

14. **There are some big pieces of work that address some of the issues you mention such as a lack of involvement of fathers in parental interventions that I might have expected to see as part of the critical analysis of findings
    (e.g. Panter-Brick, C., Burgess, A., Eggerman, M., McAllister, F., Pruett, K., & Leckman, J. F. (2014). Practitioner review: engaging fathers-recommendations for a game change in parenting interventions based on a systematic review of the global evidence. Journal of child psychology and psychiatry, 55(11), 1187-1212.)**

- On pg. 20 of the Discussion, we have added a new sentence to the paragraph about the lack of parenting interventions that engaged fathers and referenced this review by Panter-Brick et al., 2014.

15. **Summary
    I think that this is an important piece of work and like the authors I believe we need to have improved evidence and improved research. This will hopefully set the stall out for what needs to come next. I would suggest that this is a very publishable piece of research and hope that the authors will make suggested changes to improve it further.**

- Thank you for your helpful comments and support of our study.

**Reviewer #2:**

**This study seeks to synthesize findings from RCTs of parenting interventions designed to promote early child development in both high income countries and low and middle income countries through a meta-analysis. A strong rationale is provided, methods are clear, and the manuscript is very well written. Findings are compelling and will inform policy, program development, and ongoing research. As such, the study has the potential to have high impact.**

1. **I would like to see more discussion about program setting, given the heterogeneity across studies (e.g., home vising, health care), and discussion of individual vs. group-based models if possible.**

- On pg. 11, we have added a paragraph to the Results describing the variation across studies in program delivery and setting. Additionally, given this revealed heterogeneity, we conducted moderator analyses by the major types of subgroups of delivery models and setting. We did not find evidence that these factors significantly explained differences in intervention effectiveness on any of the outcomes. These new moderator results have been added as part of Tables 2 and 3.

2. **I am aware of at least two manuscripts not included that are nonetheless are highly relevant, and whose omission in each case represents a substantial limitation of the analysis:

   a. Pamela C. High, Linda LaGasse, Samuel Becker, Ingrid Ahlgren and Adrian Gardner. Literacy Promotion in Primary Care Pediatrics: Can We Make a Difference? Pediatrics April 2000, 105 (Supplement 3) 927-934. While I recognize that this is described as an alternate day randomization, the biases in the design are minimal, and the study documented important impacts of the Reach Out and Read model, which is otherwise not described in this manuscript. I would also wonder if randomization processes were sufficiently described in the articles that were included, and would suggest that this be specified/defined in the methods (i.e., study exclusion criteria page 6 - "not evaluated using a randomized controlled study design").

   b. Adriana Weisleder, Denise S.R. Mazzuchelli, Aline Sá Lopez, Walfrido Duarte Neto, Carolyn Brockmeyer Cates, Hosana Alves Gonçalves, Rochele Paz Fonseca, João Oliveira and Alan L. Mendelsohn. Reading Aloud and Child Development: A Cluster-Randomized Trial in Brazil. Pediatrics January 2018, 141 (1) e20170723; DOI:** [**https://doi.org/10.1542/peds.2017-0723**](https://doi.org/10.1542/peds.2017-0723)**. This study is a cluster RCT in a low and middle income country that documents large impacts of a group-based parenting intervention in a community setting, and it appears to have not been included because the mean age of the 2-3 year olds in the study was 37 months (vs. 36) - because it is a large study, it's omission is a significant limitation. At a minimum, I would recommend that the discussion note the exclusion of these important studies (and cite them), and perhaps note their exclusion as a limitation of the design and analyses.**

- Thank you for sharing this study by High et al., 2000. It fits our inclusion criteria, and we have now added this trial to our review. On pg. 6 of the Methods, we have clarified our exclusion criterion of a non-randomized trial design: if units (whether that is at the individual- or cluster-level) were not randomly assigned to intervention conditions (e.g., treatment versus control). Because High et al. describe their design as a randomized trial, we have new included it to our review. While this was relatively straightforward across all the included studies, we agree with the reviewer’s comment that many studies had inadequate details regarding the specific randomization processes, which contributed to a common risk of bias identified across individual articles included in our review. Per a similar comment raised by Reviewer #1, we have expanded the Limitations section of the Discussion to highlight this issue of quality and emphasize the need for improved reporting of trial methods in future parenting interventions (pg. 24).
- As the reviewer notes, the trial by Weisleder et al, 2018 was excluded for not meeting our pre-specified inclusion criteria of a sample of children younger than an average age of 36 months. The Weisleder trial focused on 2-to-4 year olds, and the sample had an average age of 37.4 months which resulted in its exclusion from our review. We do not see the omission of this study as a significant limitation, given the large number of other studies included in the review. Moreover, the results from the Weisleder trial on child cognitive, language, parenting practices, and parent-child interactions are consistent with all of the corresponding pooled effect size estimates from our review (all effects are within the 95% CI of our pooled effect size estimates suggesting robustness of our review findings).
- That being said, we have reiterated in the Limitations section (pg. 23) that our review inclusion criterion focusing on children younger than 36 months, which resulted in the exclusion of the trial by Weisleder et al. (with reference to the study) and a number of other relevant parenting interventions for young children, thus limiting the generalizability of our findings.

3. **These minor concerns aside, this is a very important addition to the literature.**

- Thank you for your helpful comments and support of our study.

**Reviewer #3: Alex McConnachie**

**This is a review of the statistical aspects of the paper by Jeong et al, a systematic review and meta-analysis of parenting interventions in the first three years of life. Overall, I found the paper to be easy to follow and interesting. Statistically, the methods that have been used appear appropriate, and are nicely presented. My comments are, in the main, relatively minor.**

1. **I note that there were 6 primary outcome domains. This multiplicity does not seem to have been taken into account in the interpretation of the results. In addition, would there have been any advantage to employing a multivariate approach, analysing primary outcomes from all six domains within the same model, allowing for correlations between intervention effects with respect to the different outcomes? This may have advantages in terms of improved estimation of pooled intervention effects, though I am certainly not an expert on these methods. The authors have done this to some extent by modelling multiple measures per domain from the same study; perhaps the potential advantages of a full multivariate analysis would not be worth the added complexity.**

- On pg. 23 of the Limitations section, we have highlighted the issue of statistical multiplicity and how caution should be applied when interpreting our results. As the reviewer notes, we have applied robust variance estimation techniques to account for multiple effect sizes per study and handle the dependency structures. This is an improved analytic strategy that has been recommended in the literature. While this method has its limitations (which we have now included on pg. 24 the Discussion), we believe it is appropriate and perhaps comparable to employing a multivariate meta-analysis approach. We agree with the reviewer that a full multivariate meta-analysis would certainly be much more complex and its added benefits beyond the robust variance estimation approach is unclear. Particularly of concern, a multivariate meta-analysis requires information about the correlations between the primary outcomes from all six ECD domains to estimate into the covariance matrix within the same model. These correlations between ECD domain outcomes were rarely reported in the individual studies and are generally unknown.

2. **On page 7, I found the phrase "studies weighted by the inverse variance method (Hedge's g)" slightly confusing. I thought Hedge's g was a way to estimate the standardised mean difference, rather than to do with the inverse variance method.**

- The reviewer is correct, and we have deleted this phrase.

3. **For the moderator analyses, was calendar date considered as a potential moderator - i.e. have effect sizes changed over time? These studies cover a 45 year period, which seems a long time, and time enough for interventions to be improved.**

- We did not consider year of publication as a potential moderator because we did not have a specific hypothesis regarding this variable. On one hand while we understand the expectation for intervention effects to improve over a 45-year period, on the other hand, the implementation approaches and types of parenting interventions have also changed substantially over time, with more of the recent programs being delivered at scale or in “lighter-touch” version that are being integrated through existing platforms and as part of other services. Therefore, this variable of calendar year is likely confounded by other trends.
- However, to at least respond to the reviewer’s question, we conducted meta-regressions using the continuous measure of publication year (range: 1974-2020) as a moderator for each ECD and parent outcome. None of the analyses indicated a statistically significant moderator effect by publication year (at *P*<0.05).

4. **One thing I thought was missing is any assessment of publication bias. Surely the authors considered this?**

- We conducted Egger’s tests to statistically assess the degree of small-study/publication bias. All of our analyses included the recommended minimum number of studies (10) to evaluate publication bias using Egger’s tests. We have added details regarding our assessment, findings, and implications of publication bias to the Methods (pg. 9), Results (pg. 12), and Discussion (pg. 24).

5. **When reporting each outcome, in the text, there is no mention of heterogeneity, which is noticeably large for some outcomes when reviewing the figures.**

- In the Results, we have included the I^2^ statistic and associated *P*-value from the Cochran *Q* test when reporting in the text the estimated pooled effect for each outcome. On pg. 16 in the first sentence of the “Moderator analyses” sub-section of the Results, we have added a sentence about how we conducted several exploratory moderator analyses given the large observed heterogeneity in nearly all the outcomes.

6. **Also, it is not clear how many studies include multiple outcomes measuring the same domain. Perhaps we could be told the number of studies included in each analysis, and the number of effect estimates used across those studies.**

- In the Results section (the first sentence of each paragraph), we now state the number of studies and the number of effect size estimates that contributed to each analysis. The forest plots (Figures 2-11) provide specific details regarding which studies provided multiple effect estimates (and for what subdomains/outcomes the different estimates correspond to).

7. **In the moderator analyses, pooled effect sizes are reported within subgroups, and described as significant, but with no p-value that I could find to support such statements. For example, on page 13, "subgroup differences for these outcomes were statistically significant (e.g., for cognitive development, SMD=0.48, 95% CI: 0.33 to 0.65 in LMICs versus SMD=0.17, 95% CI: 0.09 to 0.24)" - this shows the pooled estimates within LMICs and HICs, with 95% CIs, but there is no p-value for the difference between these two estimates. It is true that these CIs do not overlap, but that need not be the case for the difference between the subgroups to be statistically significant. This is also the case in Table 2 - we have pooled effect estimates within subgroups, but no test of differences between subgroups. As an aside, I note that Table 2 reports the LMIC estimate as 0.49, not 0.48; I suggest a thorough check of the values reported in the text versus the tables.**

- Thank you for this point. We have now included the *P*-values corresponding to the statistical tests conducted for the moderator analyses (see pg. 16-17 for the examples highlighted in text and updated Tables 2 and 3). The updated methods section on pg. 9 describes how statistical significance was assessed.
- Thank you for identifying this discrepancy in a reported estimated in text compared to the table. We have conducted a thorough check of all values reported in text versus the tables.

8. **Table 1 includes a lot of useful information, but does not give any indication of how many outcome measures each study provided in relation to each outcome domain. Rather than "X", could the table show the number of outcomes for each study within each domain?**

- Table 1 is intended to provide a high-level overview of the included studies and which of the 6 primary and 4 secondary outcomes (how many outcomes) each study measured. We believe the “X” marks in the outcomes columns are helpful in painting this general picture of what child and parent outcomes were measured across the 102 interventions. The forest plots for each analysis (Figures 2-11) indicate which studies provided more than one effect estimates (and for what subdomains/outcomes the different estimates correspond to).

9. **Returning to Table 2, for some moderators, the effect estimates relate to defined subgroups of studies, but for continuous moderators, it is not clear what the estimates and confidence intervals mean. I assume these are the estimated changes in effect estimates associated with a one-unit increase in the moderator, but I would suggest this is not very helpful. It may be better, for the table, to report effect estimates within subgroups of age and intervention duration. The statistical significance of the moderating effects of continuous measures could still be reported from a meta-regression using the continuous measure.**

- Thank you for this point. We have generated dichotomous variables for the moderators of child age and intervention duration (< or ≥12 months) and have now reported effect estimates within these two subgroups of age and duration. We have assessed statistical significance for these moderating variables based on meta-regression analyses using the continuous measures (in months). We have updated the corresponding description to the Methods section on pg. 9 and have updated Tables 2 and 3.
